# Effect of Crosslinking on the Physical Properties and Antifungal Activity of Active Coatings Containing Cinnamon Essential Oil for Anthracnose Control in Mangoes

**DOI:** 10.1111/1750-3841.70761

**Published:** 2025-12-10

**Authors:** Tamires Sousa de Oliveira, André Mesquita Magalhães Costa, Caroline Corrêa de Souza Coelho, Davy William Hidalgo Chávez, Lourdes Maria Corrêa Cabral, Otniel Freitas‐Silva, Renata Valeriano Tonon

**Affiliations:** ^1^ Food Science Graduate Program Federal University of Rio de Janeiro, UFRJ Rio de Janeiro Brazil; ^2^ Food and Nutrition Graduate Program Federal University of the State of Rio de Janeiro, UNIRIO Rio de Janeiro Brazil; ^3^ Food Science and Technology Graduate Program Federal University Rural do Rio de Janeiro Seropédica Brazil; ^4^ Embrapa Food Technology Rio de Janeiro Brazil

**Keywords:** active coating, anthracnose, cinnamon essential oil, stability

## Abstract

This study aimed to verify the stability of cinnamon essential oil (CEO) nanoemulsions after their incorporation into polymer‐based coatings for the control of anthracnose in mangoes. A 0.75% CEO emulsion with Tween 80 was produced via ultrasonication and incorporated into sodium alginate (1%) and carboxymethylcellulose (0.5%) solutions, with or without calcium‐induced crosslinking. Physical stability (droplet size, polydispersity index, zeta potential, pH, whiteness, turbidity, and antifungal activity) was monitored over 28 days. Furthermore, the rheological behavior and efficacy of the active coatings were evaluated on mangoes artificially inoculated with *Colletotrichum gloeosporioides*. Crosslinked formulation showed lower variations in droplet size and PDI over time, indicating greater protection of CEO droplets within the structured polymeric network. All coating‐forming solutions exhibited pseudoplastic behavior; however, the incorporation of the nanoemulsion reduced viscosity compared with the base coating while maintaining adequate flow behavior for brush application. Notably, the crosslinked coating achieved a substantial mitigation of the disease, reaching up to 70% reduction in anthracnose lesion progression of the mangoes. Therefore, calcium crosslinking resulted in better long‐term maintenance of physicochemical and functional properties, confirming the efficiency of this strategy in enhancing the performance of CEO‐based active coatings for sustainable postharvest disease management.

## 1 Introduction

Anthracnose is a postharvest disease caused by *Colletotrichum* spp., and it is highly prevalent in tropical fruits such as mango, banana, and papaya. The disease can cause significant economic losses due to the formation of dark lesions on fruit surfaces, which compromise appearance and marketability (Gonçalves et al. 2021). In mangoes, infection by these fungi directly impacts fruit quality and yield. Under inadequate disease management and favorable environmental conditions, postharvest losses due to anthracnose can reach up to 100% (Dofuor et al. 2023). This alarming scenario has driven increased research efforts aimed at developing strategies to reduce postharvest losses in mango production.

Although conventional treatments, particularly synthetic fungicides, are commonly used to control anthracnose, their overuse can lead to increased pathogen resistance and the accumulation of chemical residues in soil and water. These consequences pose serious risks to both human health and the environment (Bordoh et al. 2020). As a result, recent research has focused on natural alternatives, such as essential oils, for disease management in fruits and vegetables. One promising approach is the incorporation of nanoemulsified essential oils into polymer‐based edible coatings for postharvest fruit protection (de Oliveira et al. 2023).

Cinnamon essential oil (CEO) possesses well‐documented antioxidant and antimicrobial properties and has demonstrated efficacy against anthracnose in mangoes (Danh et al. 2021). However, few studies have applied nanotechnology to enhance the stability and efficiency of CEO‐based systems. In nanoemulsions (NEs), typically characterized by droplet sizes ranging from 20 to 200 nm, CEO may exhibit enhanced bioactivity due to the increased surface area and improved dispersion. Moreover, nanoemulsification can provide greater protection of CEO compounds against oxidation and increase their solubility (da Silva et al. 2022).

The use of nanoemulsified CEO enables its effective incorporation into polymeric matrices for the development of active coatings. These coatings are considered a safe, economical, and practical strategy to improve fruit quality and extend shelf life by reducing respiration rates. When essential oils are incorporated, coatings gain antimicrobial and antioxidant properties, resulting in so‐called active coatings (Mushtaq et al. 2023).

Polysaccharides, such as sodium alginate (SA) and carboxymethyl cellulose (CMC), are commonly used as polymeric bases in coating formulations due to their excellent mechanical, thermal, and chemical properties (Chettri et al. 2023). These materials also provide effective thickening and emulsion stabilization capabilities, in addition to being biodegradable, nontoxic, biocompatible, and cost‐effective (Montaser et al. 2021).

Crosslinking with multivalent cations, particularly calcium ions, has been employed to further enhance the water barrier, mechanical strength, and structural integrity of polysaccharide‐based coatings. In alginate systems, calcium‐induced crosslinking improves film cohesion and delays the release of active compounds. Although alginate films or coatings have poor moisture barriers, their hygroscopic nature helps to slow the dehydration of the coated food (Montaser et al. 2021; Morozkina et al. 2022).

Active coating systems represent complex solutions where component interactions significantly influence the final coating properties. While incorporating active compounds via nanoemulsions is widely recognized for enhancing the stability and bioavailability of natural antimicrobials, the long‐term stability and functional preservation of these complex active systems under typical storage and application conditions remain a significant technological challenge (Wibowo et al. 2024). Crosslinking the polymeric matrix, typically using calcium chloride, is a crucial strategy employed to improve the coating's mechanical barrier properties and effectively control the sustained release of active agents. Studies involving polymeric solutions and nanoemulsions have been extensively explored for various applications (Mostaghimi et al. 2023; Li et al. 2024; Gutiérrez‐Jara et al. 2021). However, few studies have specifically focused on evaluating the critical impact of calcium chloride crosslinking on the long‐term maintenance and functional preservation of the active component within the final coating structure.

In this context, the present study aims to fill this gap by developing and characterizing coating‐forming solutions based on SA (with or without crosslinking) and CMC incorporating CEO nanoemulsions. The formulations were rigorously evaluated for their physical stability and antifungal activity. Specifically, the stability of the coating‐forming solutions was assessed over 28 days, with analyses performed every 7 days for droplet size, PDI, zeta potential, pH, whiteness index (WI), turbidity, and in vitro antifungal activity. The effectiveness of the coating was also determined by applying these active coatings to mangoes artificially inoculated with *Colletotrichum gloeosporioides* in order to precisely evaluate their performance in controlling the development of anthracnose lesions.

## 2 Material and Methods

### 2.1 Material

CEO (*Cinnamomum cassia*) was obtained from Ferquima Indústria e Comércio Ltda (Vargem Grande Paulista, Brazil). SA and Tween 80 were purchased from Sigma‐Aldrich Brasil Ltda (São Paulo, Brazil), while sodium carboxymethylcellulose and calcium chloride were acquired from Êxodo Científica (Sumaré, Brazil). Glycerol was obtained from Neon Comercial (Suzano, Brazil), and the synthetic fungicide Graduate A+ was kindly provided by Syngenta Proteção de Cultivos Ltda (São Paulo, Brazil). The *C. gloeosporioides* strain used in this study was isolated from typical anthracnose lesions on mango fruits and confirmed through Koch's postulates. Fungal identification was performed by extracting genomic DNA from the fungal mycelium, followed by amplification of the internal transcribed spacer (ITS) regions of the rDNA using the primer set ITS4 (5′‐ATTAACCCTCACTAAAGTCCTCCGCTTATTGATATGC‐3′) and ITS5 (5′‐TAATACGACTCACTATAGGGGGAAGTAGAAGTCGTAACAAGG‐3′) (Ekwomadu and Mwanza, 2023). Preliminary species identification was carried out using the online tool “UNITE—rDNA ITS‐based identification of Eukaryotes and their communication via DOIs” (Kõljalg et al. 2020). Fungal isolates were cultured on potato dextrose agar (PDA) (HiMedia, Mumbai, India) at 25 ± 0.5°C for 7 days. Mangoes of the Tommy Atkins cultivar with uniform color and firmness were selected and purchased at the CEASA supply center in Rio de Janeiro, Brazil.

### 2.2 Preparation of the Active Coatings

The final formulation of the coating solution is the result of an extensive optimization effort, based on laboratory pretests, previous studies by our group (de Oliveira et al. 2024), and reference literature in the field (Manzoor et al. 2021; Ćorković et al. 2021; Zheng et al. 2019). The final concentration (w/w) of each component in the coating solutions was as follows: 0.25% CEO, 0.25% Tween 80, 1% SA (crosslinked or not with 0.75% calcium chloride), 0.5% CMC, and 20% glycerol (relative to the weight of the polymers).

The CEO nanoemulsion (NE) was prepared following the optimized conditions. A coarse emulsion, containing 0.75% (w/w) CEO, Tween 80 (1:1 w/w), and ultrapure water, was first formed by homogenization using an Ultra‐Turrax T25 at 10,000 rpm for 5 min. This coarse emulsion was then subjected to ultrasonication (34 mm diameter sonotrode, BS4d34, UIP1000hdT—Hielscher, Germany) at 150 W for 3 min. The NE was also analyzed independently to assess potential instabilities.

The polymeric solutions were prepared separately: 3% (w/w) SA and 1.5% (w/w) CMC were dispersed in ultrapure water and allowed to hydrate overnight. On the following day, the solutions were homogenized using the Ultra‐Turrax T25 at 9000 rpm for 20 min at 70°C. To evaluate crosslinking, calcium chloride (0.75% w/w based on SA weight) was added to the SA solution and homogenized at 9000 rpm for 5 min at 70°C.

The SA and CMC solutions were then combined and further homogenized at 11,000 rpm for 10 min. Glycerol (20% w/w relative to the total polymers weight) was added and homogenized for an additional 10 min. Finally, after the coating‐forming solutions reached room temperature, the NE was slowly dispersed in the polymer solutions, aided by a magnetic homogenizer, for 5 min at room temperature. The coating activated by CEO nanoemulsion (NE) and with calcium‐induced crosslinking in the alginate was named NE‐Coating Crosslinking, while the coating activated by CEO nanoemulsion without crosslinking was named NE‐Coating.

### 2.3 Characterization of NE, NE‐Coating, and NE‐Coating Crosslinking

#### 2.3.1 Particle Size Distribution, Polydispersity Index, and Zeta Potential

After 24 h of production, the samples were diluted in a 1:5 ratio, and the droplet size (*Z*‐Average), polydispersity index (PDI), and zeta potential of the NE and coating‐forming solutions were determined by dynamic light scattering (DLS) using a laser diffractometer (Zetasizer Nano ZS, Malvern Instruments Ltd., Worcestershire, UK). The DLS technique determines particle size by measuring the intensity fluctuations of scattered light caused by the Brownian motion of the particles, relating these fluctuations to the diffusion coefficient and, subsequently, to the particle size. The zeta potential of the solutions was evaluated through the electrophoretic mobility of the oil droplets in the continuous phase and expressed in mV (de Oliveira et al. 2024).

#### 2.3.2 pH

The pH of all the solutions was recorded using a Titrino plus 848 automatic titrators (Metrohm AG, Herisau, Switzerland) at 25°C (Branco et al. 2020).

#### 2.3.3 Whiteness Index and Turbidity

The WI of NE and active coating solutions was analyzed using a colorimeter (Color Spectrophotometer YS3020 ‐ Guangdong Threenh Technology Co. Ltd., Guangzhou, China). To calculate the color parameters, the illuminant D65 (daylight 6500 K), the angle of 10° for the observer, and the scale of the “CIElab” color system were established. The CIE L*, a*, and b* values were determined, and the WI was calculated with Equation (1) (Salvia‐Trujillo et al. 2015).

(1) $$WI=100\;-\left[{\left(100-L\right)}^{2}\right]+{\left(a^{2}+b^{2}\right)}^{0.5}\;$$

The turbidity of all the solutions was evaluated by absorbance measurement at 600 nm using a digital UV‐Vis spectrophotometer model UV‐M51 (Sugumar et al. 2014).

#### 2.3.4 Phase Separation Under Extreme Conditions

The accelerated stability of the solutions was evaluated under two conditions: (a) after 48 h of storage at 50°C and (b) after high‐speed centrifugation at 9000 rpm for 20 min using a Routine 38R centrifuge (Andreas Hettich, Germany). Instability was assessed based on the presence of visible changes indicative of phase separation, particularly at the top and bottom of the vials (Chu et al. 2020).

#### 2.3.5 Evaluation of the Antifungal Activity of NE, NE‐Coating, and NE‐Coating Crosslinking Against *C. gloeosporioides*

A spore suspension was prepared using a saline solution (0.9% sodium chloride) and applied to plates previously inoculated with *C. gloeosporioides* cultured for 7 days. The surface of the fungal cultures was gently scraped with a sterile inoculation loop. The resulting suspension, containing fungal fragments and spores, was filtered through sterile gauze and collected in Falcon tubes. The spore concentration was adjusted to 10⁵ spores/mL by counting under a light microscope. Aliquots of 500 µL of the NE, NE‐Coating, and NE‐Coating Crosslinking were each spread evenly onto separate PDA plates using a Drigalski spatula. Immediately afterward, a sterile 5‐mm paper disc was placed at the center of each plate and inoculated with 10 µL of the spore suspension. The plates were incubated at 25°C (±1°C) for 7 days. Fungal growth was measured using a digital caliper (Power Fix Professional). The onset of visible mycelial growth after 7 days was considered as time zero. Petri dishes containing PDA and inoculated discs without treatment served as controls. The percentage of mycelial growth inhibition was calculated according to Equation (2). All treatments were performed in quintuplicate (Aguirre‐Güitrón et al. 2022; Iñiguez‐Moreno et al. 2020).

(2) $$\%\mathrm{Inhibition}=\frac{\left(C-T\right)}{C}\;\times 100$$

where *C* is the mean diameter of the control colony (in millimeters) and *T* is the mean diameter of the treatment colony (in millimeters).

#### 2.3.6 Storage Stability

NE and the active coating solutions were stored at room temperature (28 ± 3°C), and their stability was assessed every 7 days until 28 days by monitoring droplet size, PDI, zeta potential, pH, WI, turbidity, and in vitro antifungal activity.

### 2.4 Rheological Behavior

For this comparative analysis, five samples were used: the nanoemulsion (NE); the coating (SA + CMC + glycerol); the coating crosslinking (SA crosslinked + CMC + glycerol); and the active samples, NE‐Coating and NE‐Coating Crosslinking. The rheological properties of the formulations were investigated using an Anton Paar rheometer, model MCR 510 (Anton Paar, Austria), by determining their flow curves. This analysis was performed using a plate–plate geometry with a 50 mm diameter and a gap of 0.103 mm between the plates, across a shear rate range extending from 0 to 100 s^−1^. The resulting rheograms were subsequently processed and corrected, and their data were evaluated based on empirical models, with the Herschel–Bulkley model specifically applied for curve fitting. The apparent viscosity of the solutions was determined by calculating the ratio between the shear stress and the shear rate (de Oliveira et al. 2024).

### 2.5 Application of Active Coatings in Mangoes for Anthracnose Control

The mango fruits were completely coated with the active coating solutions using a brush (Atlas, Model 319/5, 21 cm × 5.2 cm × 1 cm). The brush was applied twice, without interruption, to each side of the fruit to ensure full coverage. Subsequently, the coated fruits were air‐dried under a grid surface to eliminate any excess solution (Santos et al. 2024). Uncoated fruits and fruits with application of the fungicide Graduate A+ (prepared according to the producer's instructions) were considered as negative and positive controls, respectively. Twelve mangoes were used in each treatment. To inoculate, three injuries were made in the epidermis of each fruit, using a sterile needle (approximately 3 mm deep and 1.5 mm wide) on the same side of the fruit. Subsequently, 10 µL of the spore solution of *C. gloeosporioides* was applied to the lesions and air‐dried in a biosafety cabinet. The fruits were stored at 20 ± 1°C and 90 ± 6% of relative humidity for 10 days. The fruits were examined for visible symptoms to measure the diameter of the anthracnose lesion at the 4th, 7th, and 10th days. A storage period of 10 days was established because, during this period, the lesions on the fruits in the control group increased significantly and merged, making it impossible to quantify their size. The re‐isolation was carried out from the perceived lesions to confirm, based on the morphological characteristics of the strain, that the observed symptoms were caused by *C. gloeosporioides*. In this study, all appropriate biosafety measures were adopted. The results were presented as the percentage reduction in the diameter of the anthracnose lesion (%ALDR), in comparison to uncoated fruits (control negative), using Equation (3) (dos Passos Braga et al. 2019):

(3) $$\%\mathrm{ALDR}=\frac{N-F}{N}\;\times 100$$

where *N* represents the diameter of the lesion in the negative control and *F* is the diameter of the lesion in fruits coated with coating‐forming solutions or treated with the fungicide.

### 2.6 Data Analysis

Statistical comparisons were based on results obtained from at least three replicates, and all data are presented as mean ± standard deviation. Data were subjected to analysis of variance (ANOVA), and significant differences between means were determined using Tukey's post hoc test. All statistical analyses were performed using STATISTICA software, version 10.0 (StatSoft, Tulsa, USA), with a significance level set at *p* ≤ 0.05.

## 3 Results and Discussion

### 3.1 Droplet Size and PDI

**FIGURE 1:**
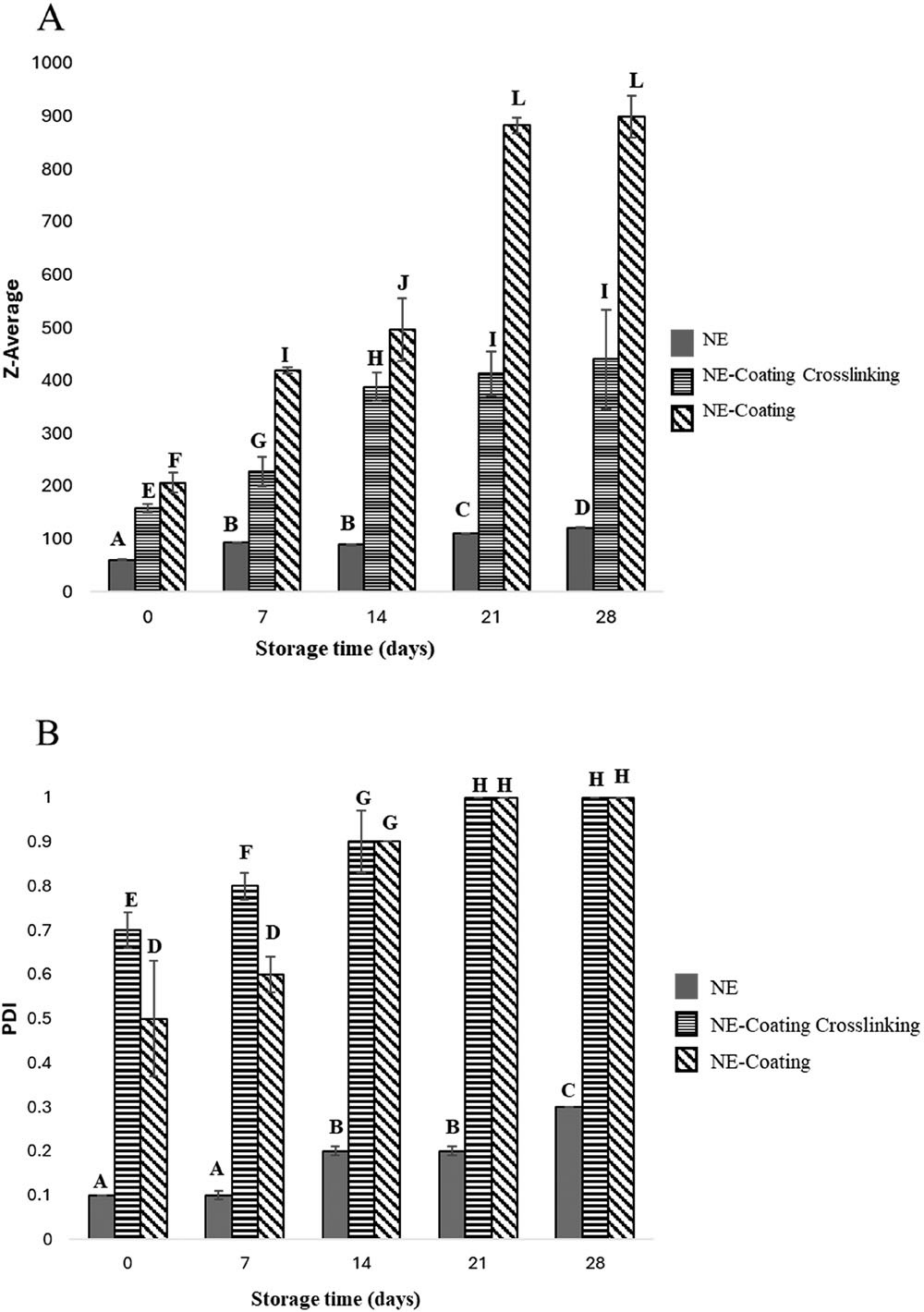
Droplet size (A) and polydispersity index (B) of NE, NE‐Coating Crosslinking, and NE‐Coating. Different uppercase letters indicate a significant difference in the same experimental run (ANOVA, *p* ≤ 0.05).

NE exhibited significantly smaller droplet size and PDI (*p* ≤ 0.05) compared to the active coating solutions throughout the storage period (Figure 1). In nanoemulsions with droplet sizes near 70 nm, gravitational forces exert minimal influence, while Brownian motion predominates, contributing to enhanced kinetic stability during storage (Sneha and Kumar 2022). A previous study by our research group showed that the pure CEO nanoemulsion had smaller droplet sizes compared to those observed after its incorporation into SA solutions (0.5% and 1%) (de Oliveira et al. 2024). Therefore, an increase in droplet size following incorporation into the coating‐forming matrix was anticipated.

A smaller variation in the average droplet size of CEO was observed in the NE‐Coating Crosslinking compared to the NE‐Coating. This trend is evident in Figure 1 and Table 1, particularly in the D50 size distribution. The structural reorganization of SA molecules induced by crosslinking with Ca^2^⁺ may have created a more favorable matrix for protecting and stabilizing the oil droplets. Chemical crosslinking involves the formation of covalent bonds that generate three‐dimensional networks, reducing the mobility of the final structure (Yang et al. 2023). Similar effects were reported by Yan et al. (2021), where alginate and zein were used to form Pickering emulsions containing curcumin and resveratrol, and crosslinking with Ca^2^⁺ resulted in a gel structure less susceptible to phase separation.

**TABLE 1:** Droplet size distribution expressed as D10, D50, and D90 of the NE, the NE‐Coating Crosslinking, and the NE‐Coating.

Storage days	Samples	D10 (nm)	D50 (nm)	D90 (nm)
0	NE	18.8^a^	64.9^a^	124.6^a^
	NE‐Coating Crosslinking	52.4^b,c^	187.7^c^	243.3^c^
	NE‐Coating	66.9^b,c^	197^c^	284.4^c^
7	NE	17.6^a^	86.6^a^	125.4^a^
	NE‐Coating Crosslinking	80.4^d^	232.3^d^	345.6^d^
	NE‐Coating	137.9^e^	460.1^f^	720.3^h^
14	NE	28.6^a^	100.7^a^	135^a^
	NE‐Coating Crosslinking	208.6^f^	399.7^e^	422.5^f^
	NE‐Coating	386.2^g^	500.6^g^	726.8^h^
21	NE	44.8^b^	105.9^a,b^	140.6^a^
	NE‐Coating Crosslinking	220.9^f^	435.5^f^	630.2^g^
	NE‐Coating	398.3^g,h^	907.7^h^	956.7^i^
28	NE	60.4^b,c^	118.2^b^	167.7^b^
	NE‐Coating Crosslinking	336.3^g^	449.3^f^	670.9^g^
	NE‐Coating	433.6^h^	928.9^h^	1,135.6^i^

*Note*: Results represent the mean ± SD (*n* = 3). Significant differences within each column are indicated by different letters (*p* ≤ 0.05) by Tukey's test.

A significant increase (*p* ≤ 0.05) in the average droplet size and PDI of CEO droplets was observed after homogenization of the NE with the polymeric coating‐forming solutions. This increase may be attributed to the absence of a high‐shear processing step during the preparation of these solutions, which could have preserved the integrity of the polymer chains, preventing depolymerization. The retention of the polymers’ native structure may result in greater steric hindrance around the oil droplets, thereby promoting the formation of larger, nonspherical droplets (Rajbanshi et al. 2024). Furthermore, the high‐shear processing can induce conformational changes in biopolymers, potentially altering their functional properties (de Oliveira et al. 2024).

The droplet radius of an emulsion, as determined by DLS, corresponds to the sum of the oil core radius and the thickness of the interfacial layer. This interface consists primarily of surfactant molecules and, potentially, polymer chains adsorbed onto the droplet surface (McClements 2011). Both coating‐forming solutions exhibited multimodal size distributions. The analysis of particle size percentiles, D10, D50, and D90, representing the droplet diameters below which 10%, 50%, and 90% of the sample population fall, respectively, offers valuable insight into the nonuniform droplet distribution within the coating matrices (Table 1). Studies reporting multimodal distributions have attributed the emergence of independent peaks to the presence of sample components such as unadsorbed surfactant micelles. Consequently, polydisperse systems may complicate the interpretation of droplet size and PDI measurements (Salvia‐Trujillo et al. 2015).

The PDI is a key indicator of emulsion quality. Values between 0.2 and 0.5 typically indicate a uniform droplet size distribution and lower susceptibility to phase separation (Shi et al. 2021). In this study, none of the samples exhibited visible signs of instability throughout the storage period. Despite this apparent stability, the PDI values of both the coating‐forming solutions were equal to or exceeded 0.5 (*p* ≤ 0.05), regardless of storage time (Figure 1). DLS analyzes the hydrodynamic radius of colloidal suspensions by detecting scattered light and converting it into intensity signals that provide insights into the sample's physical properties. However, the presence of complex matrices, including viscous, turbid, and multicomponent solutions, can compromise the sensitivity and accuracy of the technique (Rodriguez‐Loya et al. 2023).

An increase in droplet size is generally expected over the storage period, particularly when samples are stored at room temperature and thus subject to environmental fluctuations. However, coating‐forming solutions are complex polymeric nanoemulsions, containing components with diverse chemical functional groups. This chemical complexity can affect droplet size distribution, as reflected in variations in the *Z*‐average diameter. It is important to note that *Z*‐average values from DLS are unreliable when the PDI exceeds 0.5 (Instruments 2007). For example, SA‐based coating‐forming solutions containing cinnamaldehyde as an active agent have been reported to exhibit PDI values between 0.6 and 0.8, and are thus classified as polydisperse nanoemulsions (Louis et al. 2021).

### 3.2 Zeta Potential and pH

All solutions exhibited negative zeta potential values (Table 2). According to Louis et al. (2021), these negative values may be attributed to the surface charge generated by the components of CEO, particularly cinnamaldehyde, its main constituent. Furthermore, the charge observed at the oil–water interface may be enhanced by the presence of polymers, which adsorb to the surface of oil droplets surrounding the emulsifier, thereby increasing the net negative charge (Zhang and McClements 2018). Similarly, nanoemulsions formulated with CEO and lecithin have also demonstrated negative zeta potentials, which have been partially attributed to the phosphate groups in lecithin (Kaur et al. 2021). In another study, carboxymethylcellulose‐based nanoemulsions containing a mixture of thyme, cardamom, and clove essential oils exhibited negative zeta potential values, attributed to the inherently negative nature of the carboxymethylcellulose polymer (Iqbal et al. 2024).

**TABLE 2:** Stability of NE, NE‐Coating Crosslinking, and NE‐Coating in function of Zeta potential, pH, turbidity, and WI.

Tests	Samples	Storage days				
		0	7	14	21	28
Zeta potential	NE	−24.1 ± 1.2^a^	−26 ± 2.2^a^	−28.8 ± 1.26^a^	−20 ± 1.8^a^	−27.6 ± 2.4^a^
	NE‐Coating Crosslinking	−43.68 ± 1.9^b^	−49.8 ± 3.8^b^	−41.67 ± 2.6^b^	−45.76 ± 1.6^b^	−52 ± 2.2^c^
	NE‐Coating	−39.8 ± 3.7^b^	−43 ± 2.5^b^	−43.6 ± 1.6^b^	−40.7 ± 1.8^b^	−44.52 ± 2.9^b^
pH	NE	4.1 ± 0.01^a^	4 ± 0.01^a^	4 ± 0.00^a^	3.9 ± 0.06^a^	4 ± 0.05^a^
	NE‐Coating Crosslinking	6.2 ± 0.02^b^	6.1 ± 0.00^b^	6.1 ± 0.02^b^	6.1 ± 0.03^b^	6 ± 0.00^b^
	NE‐Coating	6.2 ± 0.01^b^	6.1 ± 0.00^b^	6.1 ± 0.00^b^	6.1 ± 0.00^b^	6 ± 0.00^b^
Turbidity	NE	0.7 ± 0.01^a^	1.5 ± 0.04^b, c^	2.5 ± 0.02^e^	3 ± 0.01^f^	2.9 ± 0.02^f^
	NE‐Coating Crosslinking	1.2 ± 0.04^b^	2 ± 0.01^d^	1.8 ± 0.00^c^	1.5 ± 0.00^c^	1.5 ± 0.02^c^
	NE‐Coating	1.7 ± 0.03^c^	2.1 ± 0.01^d^	1.8 ± 0.00^c^	1.9 ± 0.00^d^	1.8 ± 0.02^c^
WI	NE	53.62 ± 0.07^e^	66.96 ± 0.4^f^	70.84 ± 0.2^g^	75.73 ± 0.1^h^	76.01 ± 0.4^h^
	NE‐Coating Crosslinking	37.84 ± 0.03^c^	35 ± 0.05^b^	32.21 ± 0.2^a^	31.92 ± 0.2^a^	29.65 ± 0.1^a^
	NE‐Coating	44.45 ± 0.05^d^	38.88± 0.01^c^	35.07 ± 0.01^b^	34.94 ± 0.2^b^	29.74 ± 0.06^a^

*Note*: Samples were compared within the same experimental run, considering the entire storage period. Different lowercase letters indicate differences by Tukey's test at *p* ≤ 0.05.

It is well established that zeta potential values at or above ±30 mV are indicative of sufficient surface charge to promote electrostatic repulsion between oil droplets, thereby preventing aggregation (Rana et al. 2024). As shown in Table 2, all samples exhibited zeta potential values close to or more negative than −30 mV throughout the storage period, suggesting good colloidal stability over time. Moreover, the stability of the formulations is further supported by the fact that the zeta potential values did not differ significantly (*p* ≤ 0.05) across most time points for each individual sample.

pH is a critical parameter for assessing the stability of nanoemulsions, as it can influence particle surface charge, component solubility, and phase interactions within the emulsion. Variations in pH may lead to polymer degradation and, consequently, system instability. Moreover, pH affects the controlled release of active compounds, directly impacting antimicrobial efficacy (Singh and Nayak 2023). The pH values of the NE‐Coating and the NE‐Coating Crosslinking did not differ significantly (*p* ≤ 0.05) and remained close to neutral (approximately pH 6) over the 28‐day storage period (Table 2). This suggests that the presence of calcium chloride, the main differentiating factor between the two formulations, did not directly alter the pH. These pH values are consistent with the expected range (pH 6–7) for aqueous solutions of SA and CMC (Abka‐khajouei et al. 2022; Akbar et al. 2023; Rahman et al. 2021). The nanoemulsion maintained a stable but more acidic pH (around pH 4) during storage. Similarly, Dávila‐Rodríguez et al. (2019) reported a pH of 4.77 ± 0.01 for a CEO‐based nanoemulsion. Since pH is a key factor influencing zeta potential and, consequently, colloidal stability, the lower (in modulus) zeta potential observed in the NE compared to other samples (*p* ≤ 0.05) may be attributed to its lower pH. Colloidal suspensions tend to exhibit more positive zeta potential values at lower pH levels (Kamble et al. 2022). The consistent pH values observed in all samples throughout the storage period indicate the good physicochemical stability of these formulations.

### 3.3 Whiteness Index and Turbidity

The WI values of the coating‐forming solutions tended to decrease over the storage period, whereas droplet size showed an increasing trend (Table 2 and Figure 1). WI and turbidity are important parameters in evaluating nanoemulsions, as they directly influence the visual appearance of these colloidal systems. These optical properties are closely associated with droplet size, given that nanoemulsions with droplet sizes between 50 and 200 nm typically exhibit greater transparency or translucency (Tadros 1994). When white light interacts with an emulsion, part of it is transmitted while another portion is scattered, depending on the properties of the continuous and dispersed phases, including droplet size, size distribution, and refractive index (Salvia‐Trujillo et al. 2013).

NE exhibited increasing WI and turbidity values during storage, while both the NE‐Coating Crosslinking and the NE‐Coating showed lower values (*p* ≤ 0.05) with a slight reduction over time (Table 2). The gradual increase in droplet size observed in the nanoemulsion likely contributed to the rise in WI and turbidity, as larger droplets tend to scatter more light, resulting in emulsions with higher opacity and whiteness (McClements 2011). Moreover, the type and concentration of essential oil play a pivotal role in determining such physical properties (Salvia‐Trujillo et al. 2016; Sharifimehr et al. 2019).

NE analyzed in this study was incorporated into both the coating‐forming solutions; thus, it contained a higher concentration of CEO than the final formulations. This higher oil content may explain the significantly higher WI and turbidity values observed for the nanoemulsion compared to the other formulations (*p* ≤ 0.05). Similar findings were reported by Rao and McClements (2012), who demonstrated that increasing lemon essential oil concentration resulted in greater turbidity in nanoemulsions.

When analyzing the data collectively, a strong negative correlation was observed between WI and droplet size, with a correlation coefficient of −1 for the NE‐Coating Crosslinking (*R* ^2^ = 0.90) and −0.9 for the NE‐Coating (*R* ^2^ = 0.93) (data not shown). This supports the hypothesis that the droplet size and PDI values obtained via DLS may have been influenced by other components present in the coating‐forming formulations, as an increase in droplet size is typically associated with a more opaque, whitish appearance. The study by Sharifimehr et al. (2019) also demonstrated that the incorporation of *Aloe vera* into eugenol‐based nanoemulsions led to an increase in droplet size and a reduction in the whiteness of the coating solutions. Lower WI and turbidity are desirable in coating‐forming solutions, as they minimize alterations to the visual characteristics of the food surface. The NE‐Coating Crosslinking exhibited significantly lower WI values than the NE‐Coating (*p* ≤ 0.05), except on Day 28, when both presented similar values (*p* ≤ 0.05). This difference may be attributed to alginate crosslinking, which likely enhanced the structural integrity and stability of the oil droplets in the NE‐Coating Crosslinking, as compared to the NE‐Coating (Figure 1).

### 3.4 Visual Separation in Extreme Conditions

NE and coating‐forming solutions showed no visible signs of phase separation after being subjected to extreme conditions of gravitational force (9000 rpm) and elevated temperature (50°C). Subjecting these systems to such stress conditions can help reveal potential indicators of physical instability. The absence of phase separation under these conditions supports the droplet size, PDI, and zeta potential data obtained on Day 0 of storage, suggesting that both the NE and the coating‐forming solutions exhibit good physical stability. Similarly, Pongsumpun et al. (2020) reported no visible signs of instability in CEO nanoemulsions stored at various temperatures (4°C, 30°C, and 45°C), which aligns with the findings of the present study and further supports the stability of the emulsions evaluated.

### 3.5 Evaluation of Antifungal Activity Against *C. gloeosporioides*

The CEO has been investigated as a natural antifungal agent against various species of *Colletotrichum* spp. in several studies (Pongsumpun et al. 2020; Wang et al. 2023). In *Colletotrichum acutatum* isolated from kiwifruit, treatment with CEO resulted in damage to the cell membrane and key intracellular organelles (He et al. 2018). The same study also reported a dose‐dependent inhibition, with higher CEO concentrations leading to greater suppression of mycelial growth and spore germination.

**FIGURE 2:**
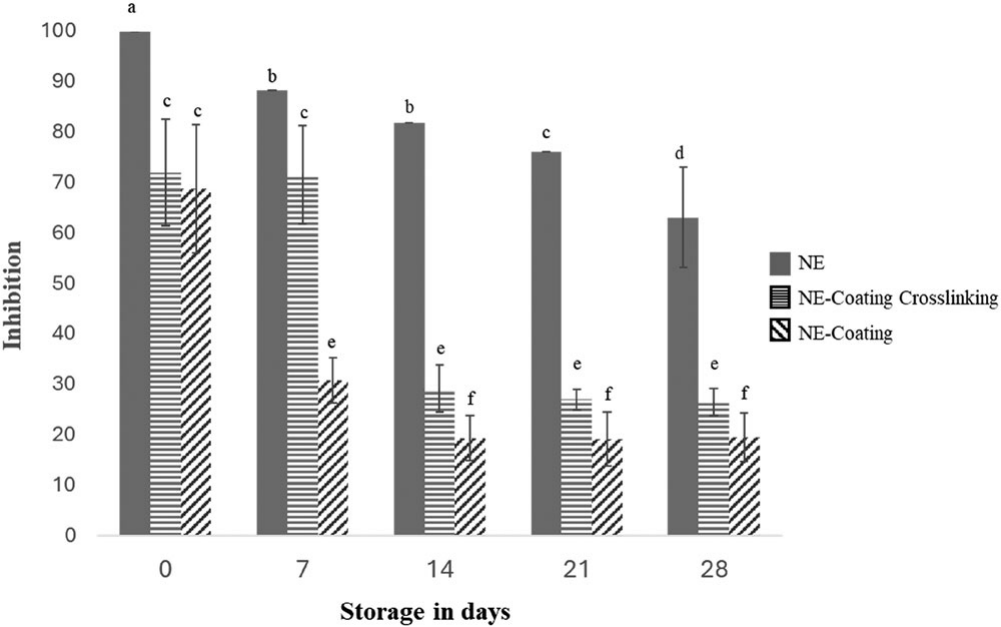
In vitro evaluation of the antifungal activity of NE, NE‐Coating Crosslinking, and NE‐Coating against *Colletotrichum gloeosporioides*.

According to Figure 2, all solutions exhibited in vitro antifungal activity against *C. gloeosporioides* over the 28‐day storage period. These findings support the previously discussed stability of the different formulations evaluated in this study. However, the NE demonstrated significantly greater antifungal activity than both the NE‐Coating Crosslinking and the NE‐Coating (*p* ≤ 0.05), as expected due to its higher essential oil content. In our previous study, a nanoemulsion containing 2% CEO produced a larger inhibition zone against *Colletotrichum* spp. compared to a nanoemulsion with 1% oil content (de Oliveira et al. 2024).

Although both the coating‐forming solutions exhibited antifungal activity throughout the storage period, the crosslinked formulation demonstrated significantly greater activity (*p* ≤ 0.05) from the seventh day onward. The crosslinking of SA with calcium ions may have resulted in a more compact polymeric network, potentially enhancing the protection and controlled release of the oil's volatile components. The NE‐Coating Crosslinking experienced a sharp decline in antifungal activity between Days 7 and 14 (*p* ≤ 0.05), after which it remained stable until Day 28. This abrupt reduction may be related to a rapid increase in droplet size observed during this same period (Figure 1).

Cinnamaldehyde, the primary component of CEO, is likely responsible for the antifungal activity observed in Figure 2. This compound possesses moderate solubility in aqueous media, which may facilitate its migration to the continuous phase, leading to release into the environment or coalescence with other droplets. This phenomenon can ultimately result in increased droplet size (de Oliveira et al. 2024). An increase in droplet size may reduce the surface interaction with microbial membranes, thereby diminishing antifungal efficacy (de Oliveira et al. 2023). In our previous study, coarse emulsions containing CEO exhibited larger droplet sizes and lower antifungal activity compared to nanoemulsions, reinforcing the correlation between these characteristics (de Oliveira et al. 2024). Additionally, CEO nanoemulsions demonstrated superior antifungal activity against gray mold and *Rhizopus stolonifer* in strawberries when compared to the pure essential oil (Naserzadeh et al. 2019).

### 3.6 Rheology

**FIGURE 3:**
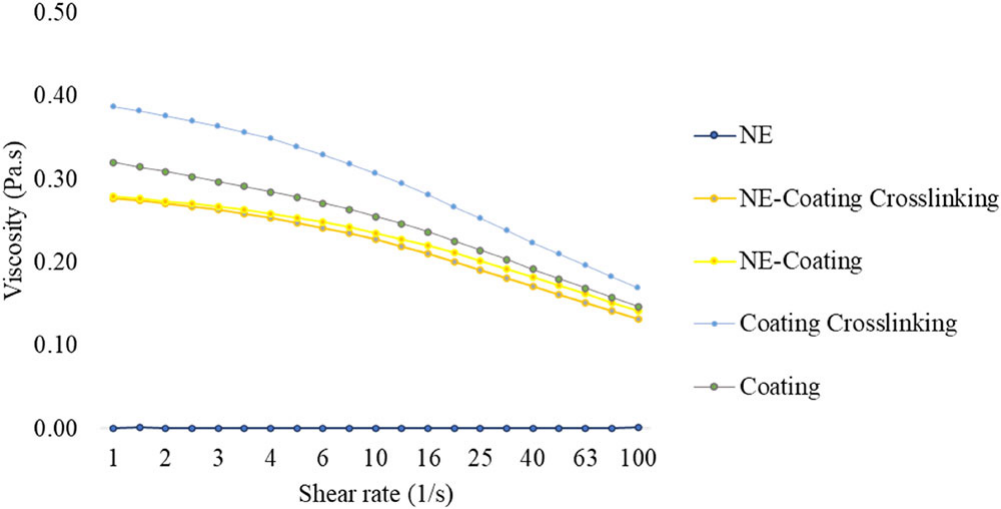
Apparent viscosity of NE (cinnamon essential oil nanoemulsion), coating (SA + CMC + glycerol), crosslinked coating (SA crosslinked + CMC + glycerol), NE‐Coating Crosslinking (NE + SA crosslinked + CMC + glycerol), and NE‐Coating (NE + SA + CMC + glycerol).

The coating‐forming solutions exhibited non‐Newtonian pseudoplastic behavior (shear thinning), which was confirmed by the decrease in apparent viscosity with increasing shear rate and by the behavior indices (*n*) being less than unity, as indicated in Table 3 and Figure 3. This rheological profile is essential for coating application, as it facilitates spreading under mechanical stress, which occurs in cases of brush application, and allows subsequent structural recovery, ensuring a stable and uniform film. NE was the only sample that exhibited Newtonian‐type fluid behavior. These data are consistent with the results obtained in our last study, in which solutions containing SA had higher viscosity than pure CEO nanoemulsions (de Oliveira et al. 2024).

**TABLE 3:** Characterization of the rheological behavior according to the consistency index (*k*) and the behavior index (*n*) of NE (cinnamon essential oil nanoemulsion), coating (SA + CMC + glycerol), crosslinked coating (SA crosslinked + CMC + glycerol), NE‐Coating Crosslinking (NE + SA crosslinked + CMC + glycerol), and NE‐Coating (NE + SA + CMC + glycerol).

Samples	Consistency index (*k*)	Behavior index (*n*)	*R* ^2^
NE	0.0003	1	0.96
Coating	0.5	0.8	0.99
Coating Crosslinking	0.6	0.7	0.99
NE‐Coating	0.4	0.8	0.99
NE‐Coating Crosslinking	0.4	0.8	0.99

The coating crosslinking presented the highest apparent viscosity and consistency index (*k*), reflecting the formation of a calcium–alginate network that restricts polymer mobility. The mild crosslinking conditions intentionally avoided full gelation, maintaining the system processable while improving structural cohesion.

The incorporation of the nanoemulsion into the NE‐Coating and NE‐Coating Crosslinking reduced viscosity relative to the base coating. This effect is associated with the plasticizing action of essential oil droplets and Tween 80, which reduce polymer–polymer interactions and enhance chain mobility, as previously reported for polysaccharide matrices containing essential oil nanoemulsions (Han et al. 2025).

However, the reduction in viscosity does not indicate an absence of ionic gelation. Several mechanisms documented in the literature help to explain this behavior. For example, Cuomo et al. (2019) explained that the nanoemulsion droplets can become partially confined within the crosslinked alginate network, decreasing their hydrodynamic contribution to the flow. Furthermore, Tween 80 can also interact with polysaccharide chains or form micellar structures that reduce the effective hydrodynamic volume of the polymer, counterbalancing the expected increase in viscosity from crosslinking with Ca^2^⁺. On the other hand, the calcium from the crosslinking agent can neutralize the negative charges of the alginate and CMC, inducing partial chain contraction and reducing intrinsic viscosity under mild gelation conditions (Rahman et al. 2021; Gradzielski 2023; Yang et al. 2023). Taken together, these effects may help explain why viscosity can decrease even when ionic crosslinking occurs. It is important to note that stability analyses showed that the crosslinked active solution obtained better results, especially in droplet size and antifungal activity, confirming the structural and functional benefits of incorporating Ca^2^⁺ and NE into the coating formulation.

### 3.7 Application as Active Coatings in Mango

**FIGURE 4:**
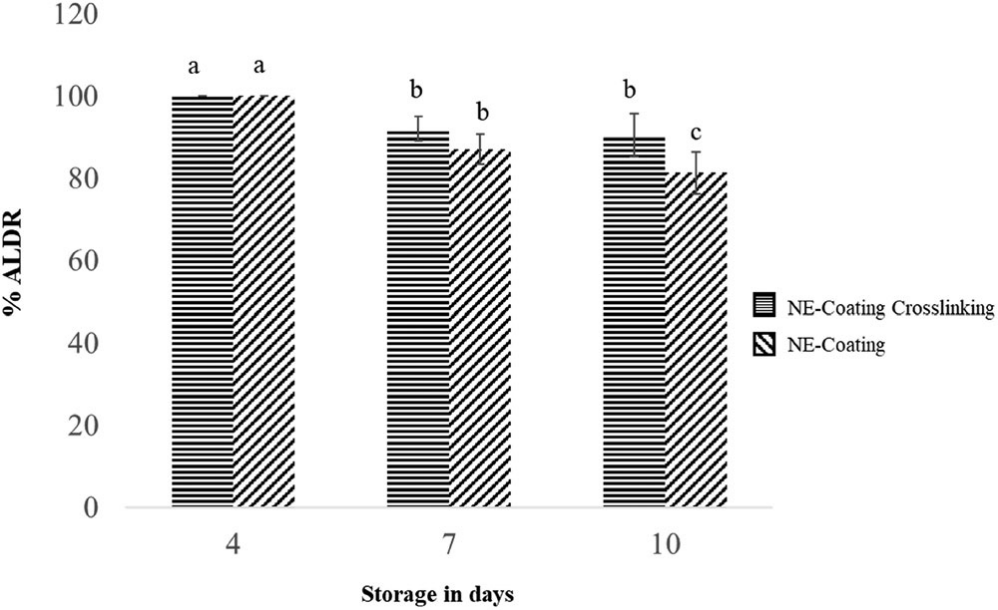
Evaluation of the antifungal activity of NE‐Coating Crosslinking and NE‐Coating on the development of anthracnose lesions in mangoes during storage expressed anthracnose lesions reduction (%ALDR).

**FIGURE 5:**
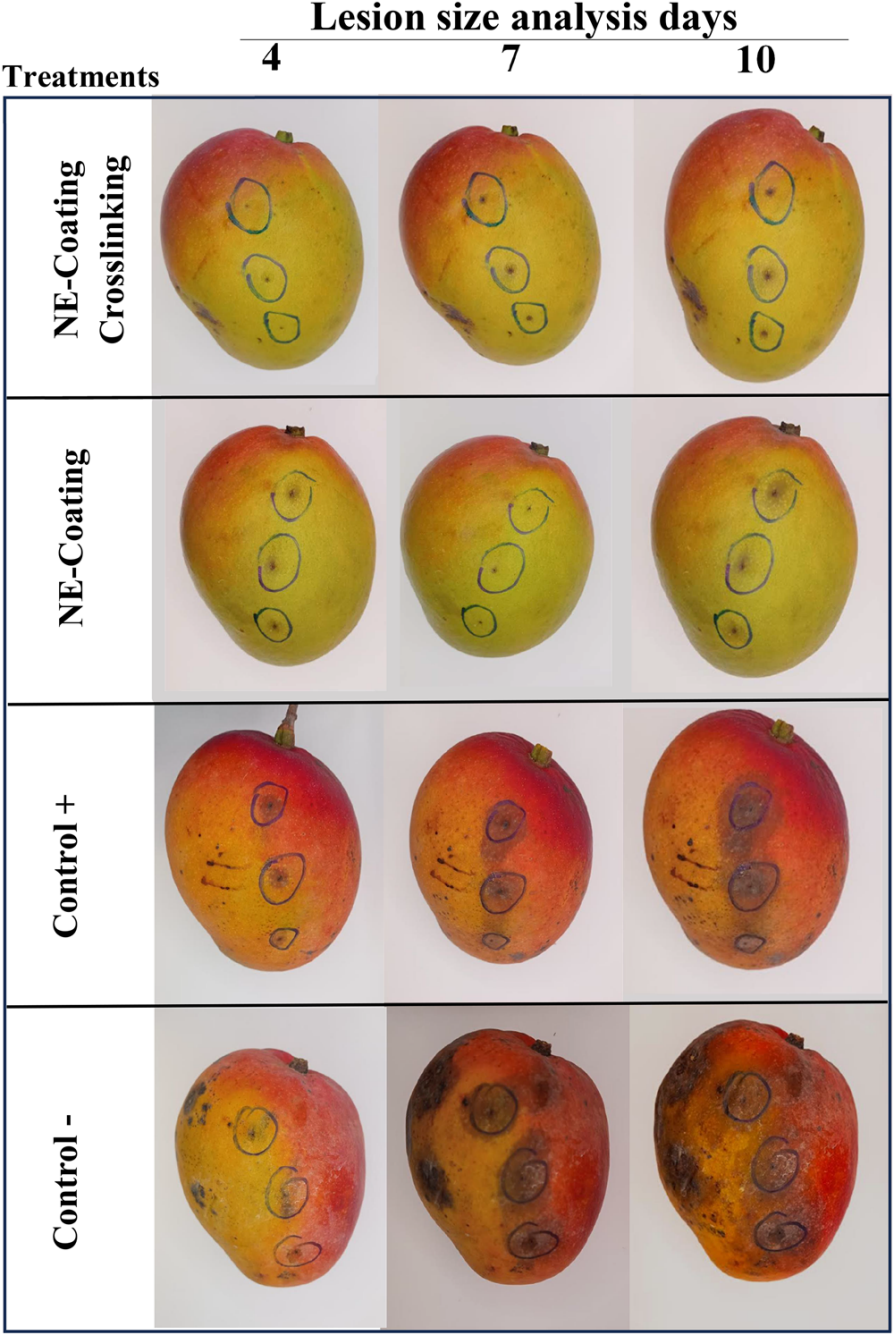
Mangoes with active coating (NE‐Coating Crosslinking or NE‐Coating) and artificially inoculated with *Colletotrichum gloeosporioides*. Control+: fruits treated with fungicide. Control‐: untreated fruits.

The evaluation of lesion size reduction in mangoes inoculated with *C. gloeosporioides* provides insights into the effectiveness of the active coatings in suppressing the disease in situ. Both the NE‐Coating Crosslinking and the NE‐Coating demonstrated strong efficacy in controlling anthracnose lesions, particularly during the first 4 days of analysis (Figures 4 and 5). Although this effect was slightly diminished over the storage period, the inhibitory activity remained substantial, with a statistically significant difference between the two coating‐forming solutions (*p* ≤ 0.05) observed only on the final day of evaluation (10 days postinoculation) (Figures 4 and 5). These findings underscore the potential of these formulations to effectively inhibit anthracnose development in mangoes.

The NE‐Coating Crosslinking exhibited a slightly greater capacity to reduce anthracnose lesions. This observation supports the hypothesis that alginate crosslinking may have enhanced the stability of the oil components, thereby improving their effectiveness in lesion reduction. Incorporating essential oils into polymeric coatings in the form of nanoemulsions can reduce the diffusion rate of volatile oil components. Additionally, such incorporation may offer better protection for essential oils against environmental factors that could compromise the functional integrity of active compounds (de Oliveira et al. 2023). For instance, various concentrations of *Mentha piperita* L. essential oil incorporated into chitosan coatings have been shown to partially or completely reduce *C. gloeosporioides* lesions in papaya during a 10‐day storage period (dos Passos Braga et al. 2019). Similarly, Shah et al. (2021) reported that mangoes inoculated with *C. gloeosporioides* and coated with chitosan containing thyme essential oil exhibited a lesion diameter of 14.8 mm on Day 9, compared to 66.25 mm in the untreated control.

Mangoes treated with the commercial fungicide (Control+) exhibited more pronounced lesions than those coated with the formulated solutions (Figure 5). The fungicide used is a commercial formulation containing azoxystrobin and fludioxonil (239 g/L*). C. gloeosporioides* isolates have demonstrated inherent tolerance to fludioxonil when evaluated for potential intrinsic resistance (Schnabel et al. 2021), which may explain the greater lesion development observed in the Control+ treatment. In this study, mangoes were artificially inoculated, a nonnatural infection scenario, which likely accelerated lesion emergence and may have contributed to the reduced efficacy of the fungicide. Moreover, the management of anthracnose in mangoes is inherently complex and often requires a multifaceted approach for effective control. Although synthetic fungicides can suppress anthracnose development, they are rarely sufficient for complete protection. Consequently, the most widely recommended postharvest strategy for controlling mango anthracnose involves an integrated approach, including preharvest eradication sprays with fungicides, fruit bagging until harvest, and postharvest hot water immersion treatments (Ciofini et al. 2022).

It is worth noting that mangoes in the control groups exhibited poorer visual appearance, particularly with respect to the artificially induced lesions. Fruits that did not receive the active coating showed more extensive damage and a more advanced stage of ripening compared to those treated with the coating‐forming solutions (Figure 5). These observations suggest that the application of active coatings can enhance mango shelf life by reducing physical deterioration and delaying ripening. These findings are consistent with those reported by Shah et al. (2021), who also developed an anthracnose control treatment for mangoes and observed a higher incidence of deterioration in untreated fruits compared to those coated with a chitosan‐based formulation activated with thyme essential oil.

## 4 Conclusion

This study successfully developed and characterized an active coating system for postharvest disease management in mangoes. The key findings and conclusions are summarized as follows:

- *Stability and structural preservation*: Crosslinking with calcium chloride was essential for improving stability over 28 days, minimizing droplet growth, and maintaining antifungal efficiency.
- *Rheological suitability for coating application*: All coating‐forming solutions exhibited pseudoplastic behavior, which is desirable for spreading by brushing. The incorporation of the nanoemulsion reduced viscosity but maintained adequate flow characteristics, ensuring practicality and homogeneous film formation.
- *Sustained antifungal performance*: The crosslinked active coating demonstrated enhanced and more stable inhibition of *C. gloeosporioides* over time in vitro, confirming superior functional preservation of the CEO.
- *Effective disease control on mangoes*: The application of the crosslinked coating reduced anthracnose development by up to 70% after 10 days of storage, surpassing the non‐crosslinked coating and performing better than the commercial fungicide under the tested conditions.
- *Final implication*: Calcium‐induced crosslinking strengthened the functionality, stability, and bioactivity of nanoemulsified CEO, confirming this formulation as a promising, safe, and sustainable alternative for extending the shelf life of mangoes and reducing postharvest losses.

Despite the increase in droplet size and polydispersity observed during storage, the crosslinked active coating remained functionally effective, maintaining antifungal activity and successfully reducing anthracnose lesion progression in mangoes. This demonstrates that the formulation is robust enough to withstand physicochemical changes without compromising its performance. However, further studies should focus on scaling this system to a pilot plant level to assess industrial feasibility, manufacturing costs, regulatory aspects, and the commercial potential of this technology. Expanding the evaluation to different mango cultivars, storage conditions, and real supply chain scenarios will be essential to validate the applicability of this eco‐friendly coating in the postharvest industry.

## Author Contributions

**Tamires Sousa de Oliveira**: conceptualization, methodology, data curation, investigation, formal analysis, visualization, writing–original draft, writing–review and editing. **André Mesquita Magalhães Costa**: conceptualization, methodology, writing–review and editing. **Caroline Corrêa de Souza Coelho**: conceptualization, methodology, writing–review and editing. **Davy William Hidalgo Chávez**: methodology, writing–review and editing. **Lourdes Maria Corrêa Cabral**: conceptualization, project administration, funding acquisition, writing–review and editing. **Otniel Freitas‐Silva**: conceptualization, methodology, visualization, writing–review and editing, project administration, resources. **Renata Valeriano Tonon**: conceptualization, supervision, project administration, resources, visualization, funding acquisition, writing–review and editing.

## Funding

This work was supported by CAPES (Grant number: 001), FAPERJ (Grant numbers: E‐26/202.710/2019, E‐26/202.325/2019, and E‐010.100994/2018), and CNPq (Grant number: 311529/2021‐6).

## Conflicts of Interest

The authors declare no conflicts of interest.

## Data Availability

No datasets were generated or analyzed during the current study.
